# 290. Outcomes of Orthotopic Liver Transplant Recipients with Bacteremia Shortly Before Transplantation

**DOI:** 10.1093/ofid/ofaf695.093

**Published:** 2026-01-11

**Authors:** R Alfonso Hernandez Acosta, Deirdre Axell-House, Satish Mocherla, Elizabeth R Ramos Salazar, Jiejian Lin, Ashton Connor, Constance M Mobley, Kevin Grimes, R Mark Ghobrial, Cesar A Arias, Masayuki Nigo

**Affiliations:** SSM Health / St. Louis University, St. Louis, MO; Houston Methodist Hospital / Houston Methodist Research Institute, Houston, Texas; Houston Methodist Hospital, Houston, Texas; Houston Methodist Hospital, Houston, Texas; Houston Methodist Hospital, Houston, Texas; Houston Methodist Hospital, Houston, Texas; Houston Methodist Hospital, Houston, Texas; Center for Cell and Gene Therapy, Baylor College of Medicine, Texas Children’s Hospital, Houston Methodist Hospital, Houston, Texas; Houston Methodist Hospital, Houston, Texas; Houston Methodist and Weill Cornell Medical College, Houston, TX; Houston Methodist Hospital, Houston, Texas

## Abstract

**Background:**

In clinical practice, bloodstream infections (BSIs) may delay elective transplantation until a “traditional” treatment course of up to 14 days is finished. However, there is a scarcity of data about the outcome of recipients that require urgent orthotopic liver transplantation (OLT) within 14 days of bacteremia.

**Table 1:**
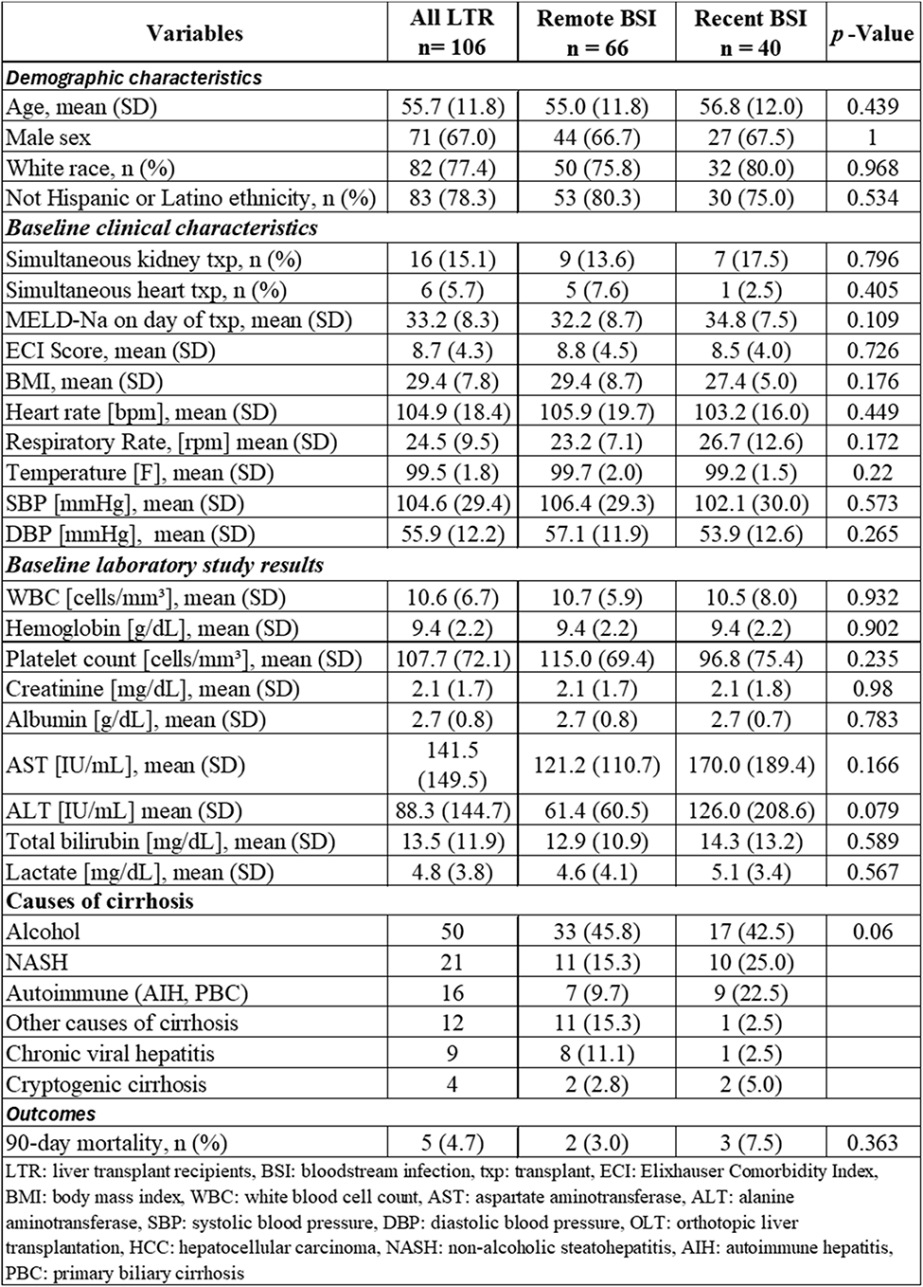
Demographic, clinical, laboratory characteristics, and outcomes.

**Table 2:**
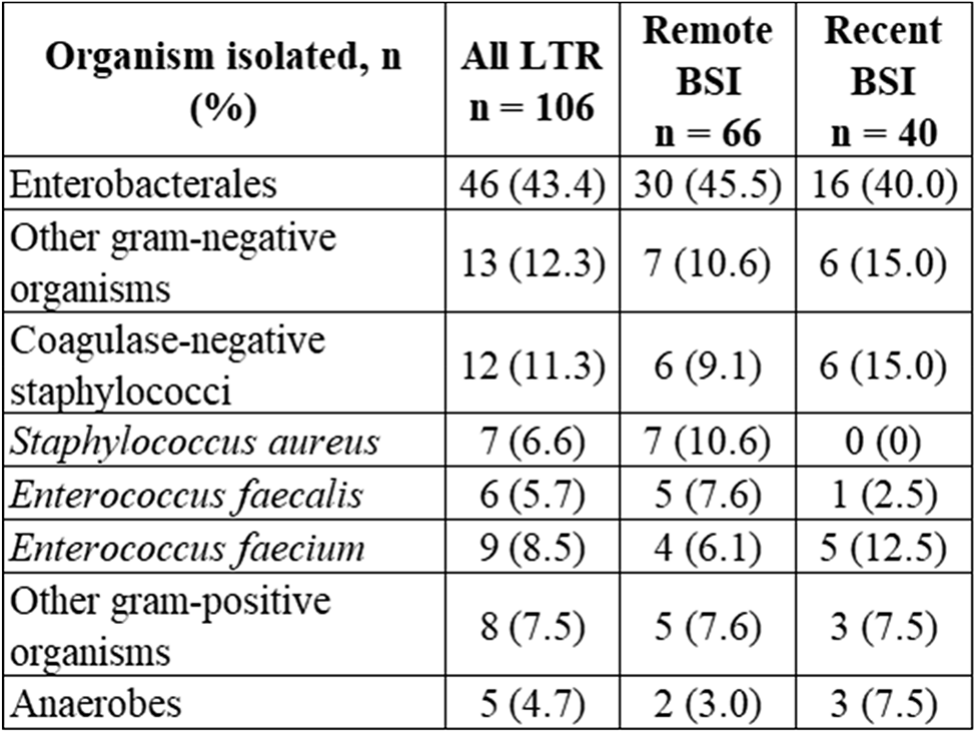
Organisms isolated on blood cultures of study subjects.

**Methods:**

A retrospective observational study was performed that included liver transplant recipients (LTR) at Houston Methodist Hospital that underwent OLT between 7/16/2016 and 7/16/2023 who had positive blood cultures in the 90 days before OLT. Data on demographics, clinical course, and outcomes were collected. Outcomes were compared between LTRs with bacteremia within 14 days (recent BSI) and more than 14 days (remote BSI) before OLT.

**Results:**

A total of 106 LTR were identified in the study period, with 40 LTR having recent BSIs. Most LTR were Non-Hispanic/Latino (75% and 80%, p=0.53) and male (67% and 70%, p=1.0) with a mean age of 56.8 and 55.0 years in recent and remote BSI, respectively. [Table 1] The most common indication for OLT was alcoholic cirrhosis. Enterobacterales (particularly Escherichia coli and Klebsiella pneumoniae) were the most common pathogens causing bacteremia and no Staphylococcus aureus bacteremia was found in the recent BSI group. [Table 2] Within 1 year of OLT, LTR with recent BSI had less recurrent BSI or clinically apparent infection with the same organism that caused bacteremia than remote BSIs. 3 LTRs (7.5%) and 2 LTRs (3%), p=0.36 in recent vs. remote BSI groups, respectively died within 90-days post-OLT. Notably, in recent BSI group, two out of the three LTRs who died within 90 days post-OLT had bacteremia with vancomycin-resistant Enterococcus faecium (VRE).

**Conclusion:**

In our study, LTR who had recent BSI did not have worse outcomes compared to those LTRs who waited longer to undergo OLT and had no recurrent infection at 1-year post-OLT. This suggests that potential recipients with bacteremia, except for VRE and S. aureus, may be considered for urgent transplantation before completing a full 14-day treatment, provided that a thorough risk and benefit assessment is conducted. A limited sample size and potential selection bias should be acknowledged, as potential recipients with bacteremia who were too sick to undergo OLT were not evaluated.

**Disclosures:**

Cesar A. Arias, MD/PhD, UptoDate: Royalties

